# Endoplasmic reticulum stress induced by turbulence of mitochondrial fusion and fission was involved in stressed cardiomyocyte injury

**DOI:** 10.1111/jcmm.17901

**Published:** 2023-08-18

**Authors:** Shengnan Zhang, Yingmin Li, Weihao Zhu, Lihua Zhang, Lei Lei, Xiaofei Tian, Ke Chen, Weibo Shi, Bin Cong

**Affiliations:** ^1^ Department of Forensic Medicine Hebei Medical University, Hebei Key Laboratory of Forensic Medicine, Collaborative Innovation Center of Forensic Medical Molecular Identification Shijiazhuang China

**Keywords:** endoplasmic reticulum stress, mitochondrial fission, mitochondrial fusion, myocardial injury, stress

## Abstract

Mitochondria are sensitive organelles that sense intrinsic and extrinsic stressors and maintain cellular physiological functions through the dynamic homeostasis of mitochondrial fusion and fission. Numerous pathological processes are associated with mitochondrial fusion and fission disorders. However, the molecular mechanism by which stress induces cardiac pathophysiological changes through destabilising mitochondrial fusion and fission is unclear. Therefore, this study aimed to investigate whether the endoplasmic reticulum stress signalling pathway initiated by the turbulence of mitochondrial fusion and fission under stressful circumstances is involved in cardiomyocyte damage. Based on the successful establishment of the classical stress rat model of restraint plus ice water swimming, we measured the content of serum lactate dehydrogenase. We used haematoxylin–eosin staining, special histochemical staining, RT‐qPCR and western blotting to clarify the cardiac pathology, ultrastructural changes and expression patterns of mitochondrial fusion and fission marker proteins and endoplasmic reticulum stress signalling pathway proteins. The results indicated that mitochondrial fusion and fission markers and proteins of the endoplasmic reticulum stress JNK signalling pathway showed significant abnormal dynamic changes with the prolongation of stress, and stabilisation of mitochondrial fusion and fission using Mdivi‐1 could effectively improve these abnormal expressions and ameliorate cardiomyocyte injury. These findings suggest that stress could contribute to pathological cardiac injury, closely linked to the endoplasmic reticulum stress JNK signalling pathway induced by mitochondrial fusion and fission turbulence.

## 1 INTRODUCTION

Stress is a pervasive component of life that affects all individuals, with different intensities and frequencies. Epidemiological studies have indicated that stress is an important cause of the development of mental diseases. About 80%–90% of depressed patients have experienced acute stressful events before the onset of the disease.^1^, ^2^ Stress elicits increases in sympathetic activity and hypothalamic–pituitary–adrenocortical (HPA) axis responses that lead to changes in cardiac function and vascular resistance, with the consequent redistribution of blood flow. While there are individual differences in coping with stress, many studies indicate that chronic stress has important pathological consequences for the cardiovascular system. Stress increases the prevalence and severity of cardiovascular diseases, such as hypertension, diabetes, obesity, and so on.^3^, ^4^ In addition, stress directly leads to cardiac injury, resulting in myocardial ischemia and remodelling.^5^, ^6^ However, the pathological mechanisms responsible for myocardial damage during stress response remain unclear.

The mitochondrion is a sensitive organelle that senses and responds to intrinsic and extrinsic stressors and sustains body metabolic requirements through morphological alterations. Mitochondrial morphology is mainly regulated by mitochondrial fusion and fission and determines mitochondrial function under strict quality control.^7^, ^8^ Mitochondrial fusion is composed of outer membrane fusion regulated by mitofusin 1 (Mfn1) and mitofusin 2 (Mfn2) and inner membrane fusion regulated by optic atrophy protein 1 (Opa1), which enables the exchange of mitochondrial DNA (mtDNA), proteins, lipids, and metabolites and mediates functional complementation through content mixing to maximize the cellular response to external stimuli.^9^, ^10^ In addition, mitochondrial fusion also enhances the integrity of the endosomal cristae to promote oxidative phosphorylation and increase (adenosine triphosphate) ATP production, thus meeting the energy metabolism demands of different types of cells.^8^, ^11^ Mitochondrial fission is mainly regulated by dynamin‐related protein 1 (Drp1) and its ligand Fis1. Moderate fission facilitates the removal of damaged mitochondria to maintain cellular homeostasis, while excessive fission leads to cellular injury.^12^, ^13^ Under physiological conditions, mitochondria fuse into tubular networks against external stimuli and maintain dynamic homeostasis, whereas excessive stimuli disturb the dynamic equilibrium, mitochondrial fusion decreases and mitochondrial fission increases accordingly. Of note, accumulating evidence indicates that mitochondrial dysfunction due to the turbulence of mitochondrial fusion and fission is associated with the development of numerous diseases.^14^, ^15^ Mitochondria are highly dynamic organelles that interact with other organelles, such as the endoplasmic reticulum, to communicate information. Previous studies have indicated that the tight contact sites formed between mitochondria and the endoplasmic reticulum–mitochondrial endoplasmic reticulum microregions have critical regulatory functions in cellular biological activities, including energy metabolism and promotion of cell death processes.^16^ Therefore, once cellular stress results in the turbulence of signalling between the mitochondria and endoplasmic reticulum, it can initiate an endoplasmic reticulum stress response that affects cellular proteins, lipid synthesis, and post‐translational modifications, further determining cell fate.

Given that cardiomyocytes, as the power source of the body, are bound to contain a large number of mitochondria, we hypothesized that stress could lead to the turbulence of mitochondrial fusion and fission and activate endoplasmic reticulum stress response to participate in the injury process of cardiomyocytes. To verify this hypothesis, we detected changes in cardiomyocyte injury. We investigated the dynamic changes in mitochondrial fusion, fission, and endoplasmic reticulum stress‐related proteins in the context of successfully establishing a rat model of daily restraint for 8 h and forced ice‐water swimming for 5 min. These findings contribute to further clarification of the molecular mechanisms underlying cardiomyocyte injury.

## 2 MATERIALS AND METHODS

### 2.1 Animal

Healthy adult male Sprague Dawley (SD) weighing between 200 ± 20 g was purchased from Beijing Huafukang Biotechnology Co. Ltd., China. The rats were housed in groups (*n* = 4/cage) in a temperature‐ and humidity‐controlled room with a 12 h light–12 h dark cycle. Food and water were provided ad libitum. The rats were randomly divided into the control group and restraint stress combined with ice water swimming (RSIS) group at 3, 7 and 21 days (*n* = 12/group). In addition, to investigate the effects of mitochondrial fusion and fission on cardiomyocytes under stress exposure, we established a group of rat models with restraint stress combined with ice water swimming for 7 days (RSIS), a group exposed to stress that was treated with Mdivi‐1 (M1), which can stabilize mitochondrial fusion and fission for 7 days (RSIS + M1), a group that was only treated with Mdivi‐1 for 7 days (M1), and a control group (*n* = 12/group). All procedures followed the National Institutes of Health guidelines and were approved by the Hebei Medical University Institutional Review Board for Animal Experiments.

### 2.2 Animal treatments

Restraint stress and ice‐water swimming tests were performed as previously described.^17^ Briefly, rats were placed in a restraint device with no access to food or water for 8 h (from 8:00 AM to 4:00 PM) each day. The restrained rats were placed in ice water to swim for 5 min each day. The process lasted for 3, 7 and 21 days. The rats in the control group were left in cages for the same amount of time with no food or water. For the RSIS + M1 group, rats were injected intraperitoneally (i.p.) with Mdivi‐1 (10 mg/kg i.p.) half an hour before stress treatment. For the M1 group, rats were injected with Mdivi‐1 (10 mg/kg, i.p.). The protocol for the RSIS+M1 and M1 groups was performed for 7 days.

### 2.3 Serum lactate dehydrogenase (LDH) test

Additionally, the rats were anaesthetised with 2% pentobarbital sodium intraperitoneally, and blood was collected by cardiac puncture. The samples were centrifuged at 4°C, 1000*g* for 15 min and the supernatant was collected and stored at −80°C. LDH ELISA Kits (Hcusabio, CSB‐E11324r) were used to measure serum LDH levels in the different groups. A rat‐derived lactate dehydrogenase (LDH) ELISA kit was used to determine the serum levels of lactate dehydrogenase according to the manufacturer's instructions. Briefly, setting up standard wells and sample wells, 50 μL of standards of different concentrations are to be added to each of the standard wells, and 50 μL of samples are to be added to the sample wells; blank wells are not added, except for the blank wells, 100 μL of HRP‐labelled detection antigen to be added to each of the standard wells and sample wells, and incubated the wells in a 37°C thermostat for 1 h. They were then washed five times with washing buffer. Subsequently, each well‐added 50 μL of substrate A, and substrate B was incubated for 15 min at 37°C, avoiding light. Add 50 μL of termination solution to each well, and the OD value of each well was measured at 450 nm within 15 min.

### 2.4 Haematoxylin–eosin (HE) staining and chromotrope‐2R brilliant green staining

The isolated rat heart was fixed in 10% formalin, and the tissue was subsequently dehydrated in steps of ethanol and embedded in paraffin. The heart wax block was sectioned into continuous sections (5 μm) for haematoxylin and eosin (HE and Chromotrope‐2R brilliant green staining. Chromotrope‐2R brilliant green staining is a sensitive method to assess cardiomyocyte injury.^18^, ^19^ Slices were stained with Chromotrope‐2R brilliant green staining for 10 min, fractionated three times in 2R fractionation solution for 1 min each, re‐stained in bright green staining solution for 10 min and finally observed under a light microscope (Olympus IX71; Mt. Olympus, Tokyo, Japan).

### 2.5 The changes in mitochondria morphology

The isolated rat heart tissue was immediately placed in an icebox to obtain a volume of 1 × 1 × 4 mm^3^ of ventricular tissue, fixed in 2.5% glutaraldehyde at pH 7.4 and delivered to the Electron Microscopy Laboratory, Hebei Medical University. The heart tissue was dehydrated in acetone at different gradients and embedded in epoxy resin. At least 400 mitochondria were randomly selected from five regions in each sample. Image J software was used to analyse mitochondrial morphology changes and cross‐sectional areas (mitochondrial cross‐sectional integration categories were classified according to three ranges of <0.6 μm^2^, 0.6–1.0 μm^2^, >1.0 μm^2^).^20^, ^21^ Further mitochondrial analysis was performed by calculating the percentage of mitochondria in a given field that fell into these three size categories.

### 2.6 Examination of reactive oxygen species (ROS) level

The rat heart was immediately isolated and snap‐frozen in liquid nitrogen and then stored at −80°C. The tissue was cut into frozen sections (9 μm) and restored to room temperature for moisture control. Tissues were incubated with a spontaneous fluorescence‐quenching agent for 5 min and washed for 10 min. The ROS probe (Sigma‐Aldrich) was added, incubated at 37°C for 30 min in the dark and washed three times with PBS (pH 7.4) for 5 min × 3 times. Finally, an anti‐fluorescent quenching agent was added. Five areas were selected randomly from each sample. ImageJ software was used to evaluate ROS levels' relative average fluorescence intensity.

### 2.7 RNA extraction and RT‐qPCR

Total mRNA was extracted from heart tissue using TRIzol (Invitrogen) and chloroform plus isopropanol precipitation. An RNA‐PCR Reverse Transcription Kit (RR047A; Takara Bio) synthesized cDNA. Quantification of Drp1, Fis1, Cyto‐c, Mfn1, Mfn2, Opa1, IRE1‐α, (apoptosis signalling‐regulated kinase 1) ASK1, JNK mRNA of rat heart tissue through SYBR Green RT‐PCR kit (RR820A; Takara Bio). Expression of the target gene was mainly calculated by 2^−ΔΔct^ normalisation of the glyceraldehyde 3‐phosphate dehydrogenase (GAPDH) gene.

The sequences of the primers are as follows:

GAPDH: Forward primer: 5′‐ AGGGCTGCCTTCTCTTGTGAC‐3′;

Reverse primer: 5′‐ TGGGTAGAATCATACTGGAACATGTAG‐3′.

Mfn1: Forward primer: 5′‐ CCATCACTGCGATCTTCGGCCA‐3′;

Reverse primer: 5′‐ CAGCGAGCTTGTTTCTGTAGCCCT‐3′.

Mfn2: Forward primer: 5′‐ GGACCTGAATCGGCACAGAG‐3′;

Reverse primer: 5′‐ GAGCAGGGACATCTCGTTTC‐3′.

Opa1: Forward primer: 5′‐ CAGCTGGCAGAAGATCTCAAG‐3′;

Reverse primer: 5′‐ CATGAGCAGGATTTTGACACC‐3′.

Drp1: Forward primer: 5′‐ TGACATCTTGACCGCCATTA‐3′;

Reverse primer: 5′‐ TGGGCTCCTCTAGACGCTTA‐3′.

Fis1: Forward primer: 5′‐ GTAGGGTTACATGGATGCCCAGAGA‐3′;

Reverse primer: 5′‐ GGCAAAAGCTCCTCCAGCAG‐3′.

Cyto‐c: Forward primer: 5′‐ GGCAAGCATAAGACTGGACCAA‐3′;

Reverse primer: 5′‐ TTTCCAAATACTCCATCAGGGTATC‐3′.

IRE1‐α: forward primer: 5′‐ TGGACGGACAGAATACACCA‐3′;

Reverse primer: 5′‐ TGGACACAAAGTGGGACATC‐3′.

ASK1: Forward primer: 5′‐ GAGGCCAAGGCGTTCATACT‐3′;

Reverse primer: 5′‐ AAGATACTCCTCTCCGTGCAAC ‐3′.

JNK: Forward primer: 5′‐ CAAGGAGGTCATGGATTTGG −3′;

Reverse primer: 5′‐ AAGACGACGGATGCTGAGAG −3′.

### 2.8 Western blot

Rat heart tissues were homogenized mechanically with 10× tissue lysis RIPA buffer (Abcam, AB156034) containing a protease inhibitor cocktail (Report, RP‐WA0120, China) and a phosphatase inhibitor (Report, RP‐WA0130, China). After centrifugation at 4°C for 14,000 *g*, 15 min, the protein concentration was measured using a BCA quantification kit (Thermo Fisher Scientific, A53225). Additionally, proteins were separated using an 8%–12% polyacrylamide gel SDS‐PAGE (ZomanBio, ZD304C) kit. Electrophoresis transferred proteins to polyvinylidene fluoride membranes (PVDF) (Beyotime, FFP33, FFP24). Bio‐rad Transblot was used to isolate the antibody GAPDH (1:1000) (Beyotime, AG019) with 5% skim milk at 37°C for 1 h including Drp1 (1:1000) (Abcam, ab184247); Drp1^ser616^ (1:1000) (Affinity, AF8470); Fis1 (1:1000) (ABclonal, A19666); Mfn1 (1:1000) (Proteintech, 13,798‐1‐Ap); Mfn2 (1:1000) (Proteintech, 12186‐1‐AP); Opa1 (1:1000) (Proteintech, 13798‐1‐AP); Cyto‐c (1:1000) (Huaan, ET1610‐16); IRE1‐α (1:1000) (Proteintech, 27528‐1‐AP); ASK1 (1:1000) (Proteintech, 28201‐1‐AP); JNK (1:1000) (Proteintech, 66210‐1‐lg). Furthermore, the PVDF membrane was incubated with the corresponding fluorescent secondary antibody (Rockland, USA) at 37°C, away from light, for 1 h. Specific protein bands were visualized with Azure C500 (Azure Biosystems). The ImageJ software quantified and analysed protein bands (NIH).

### 2.9 Statistical analysis

All experiments were independently performed at least three times. All data were collected as the mean ± SD and analysed using SPSS 21.0 (IBM SPSS Statistics). One‐way or two‐way analysis of variance (anova) and Tukey's post hoc test were used to analyse data for comparisons between groups. Statistical significance was set at *p* < 0.05 was considered significant.

## 3 RESULTS

### 3.1 Stress resulted in cardiomyocytes injury

**FIGURE 1:**
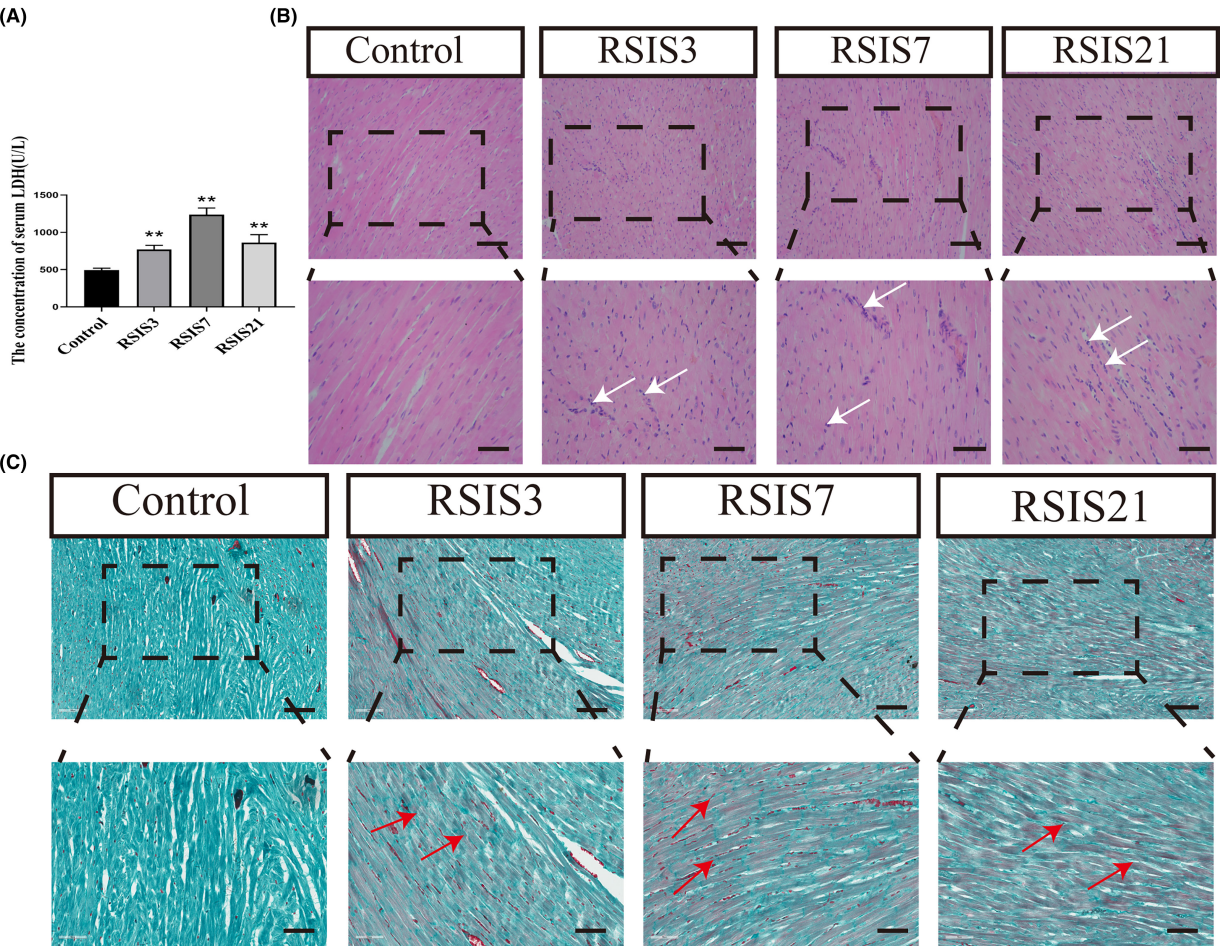
Stress resulted in cardiomyocyte injury. (A) Serum LDH changes after stress exposure. Data were presented as mean ± SD, **p < 0.01 compared with control, *n* = 4. (B) Haematoxylin–eosin staining (inflammatory cells, white arrows). (C) Chromotrope‐2R brilliant green staining (ischemia and hypoxia, red arrows). Scale bars = 100 μm or 50 μm. There was an insignificant difference between the control group with time (not shown).

We first established a stress model to investigate stress's effects on rat cardiomyocytes. In line with previous studies,^22^ we confirmed the success of the models by investigating changes in rat body weight, norepinephrine (NE) content, behaviour and stool count (Figure S1). We examined the effects of stress on LDH, a biochemical marker of cardiomyocyte injury. One‐way anova for the concentration of serum LDH showed a significant effect after stress exposure (*F*_(4,16)_ = 66.22, *p* < 0.001). Post hoc comparisons showed that LDH levels were significantly enhanced after 3 days (*p* < 0.01), 7 days (*p* < 0.01) and 21 days (*p* < 0.01) of stress exposure (Figure 1A). In addition, we examined the effect of stress on cardiomyocyte injury from a morphological perspective using traditional HE and Chromotrope‐2R brilliant green staining. As shown in Figure 1B,C, HE staining indicated that with the prolongation of stress exposure, cardiomyocytes were oedematous and eosinophilic, and inflammatory cells could be found in the myocardial interstitium. Chromotrope‐2R brilliant green staining can stain cardiomyocytes with red ischemia and hypoxia. Consistent with the HE results, cardiomyocytes exhibited noticeable ischemic and hypoxic changes during prolonged stress exposure.

### 3.2 Stress disturbed the dynamic balance of mitochondrial fusion and fission

**FIGURE 2:**
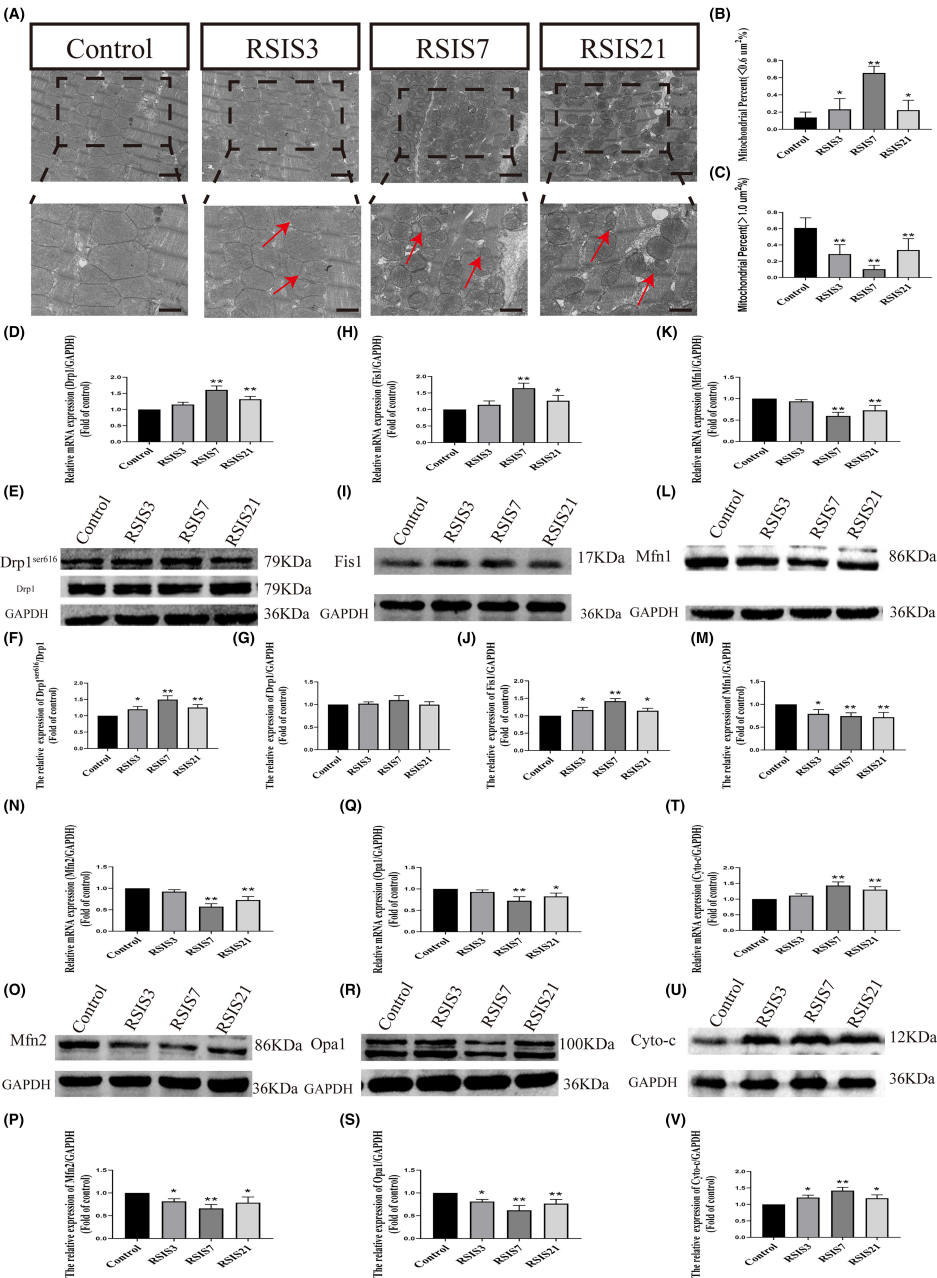
Stress disturbed the dynamic balance of mitochondrial fusion and fission (mitochondria fission, red arrows). (A) Ultrastructural changes of mitochondria in cardiomyocytes. Scale bars = 2.0 μm or 1.0 μm. Quantification of mitochondria with size. The analysis of mitochondria was achieved by calculating the percentage of mitochondria in a given field that fell into three size categories based on area: <0.6 μm^2^, 0.6 μm^2^–1.0 μm^2^ and >1.0 μm^2^ (B, C). Quantification of mRNAs and proteins of mitochondrial fission and fusion (D–V). Stress significantly inhibited mitochondrial fusion and increased mitochondrial fission. Data were presented as mean ± SD, *p < 0.05, **p < 0.01 compared with control, *n* = 4. There was an insignificant difference between the control group with time (not shown).

**FIGURE 3:**
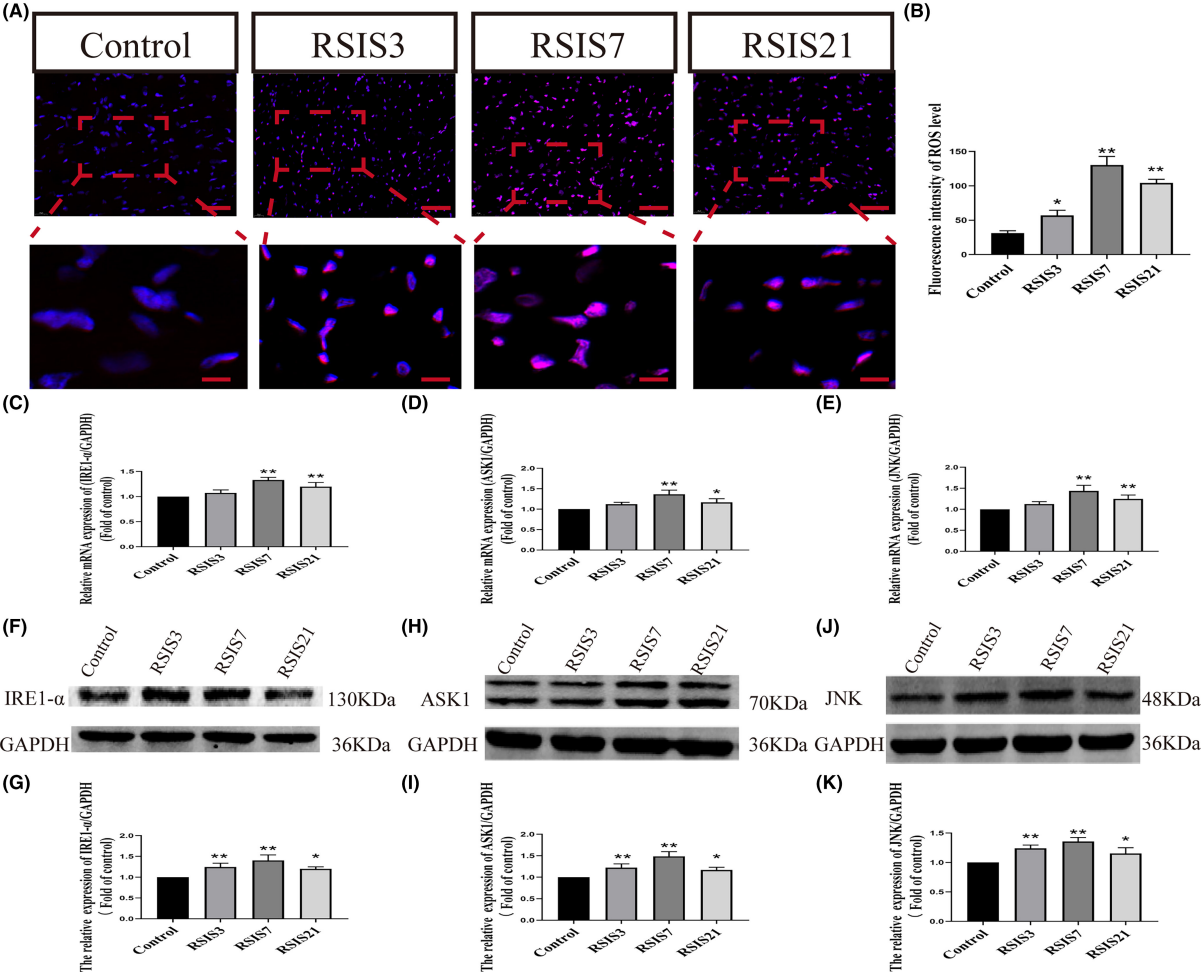
Mitochondrial dysfunction activated endoplasmic reticulum stress JNK pathway. (A, B) Quantification of intracellular ROS level after stress exposure. Scale bars = 20 μm. (C–K) Quantification of mRNAs and proteins of endoplasmic reticulum stress JNK pathway after stress exposure. Data were presented as mean ± SD, *p < 0.05, **p < 0.01 compared with control, *n* = 4. There was an insignificant difference between the control group over time (not shown).

Mitochondria are mainly divided into submembrane, perinuclear and intermyofibrillar mitochondria. According to previous research, sarcoplasmic mitochondria have a higher oxidative capacity than submembranous mitochondria^23^, ^24^ and are most closely associated with oxidative phosphorylation levels. Therefore, we selected mitochondria from the sarcoplasm for an in‐depth investigation. We observed the ultrastructural changes in the mitochondria of cardiomyocytes using electron microscopy. After stress exposure, more mitochondria were observed in the heart per field of view (FOV). In addition, the mitochondria were smaller under stress conditions when compared to control hearts, as evidenced by the reduced areas and perimeters (Figure 2A). Further analysis of mitochondria was achieved by calculating the percentage of mitochondria in a given field that fell into three size categories based on area: <0.6 μm^2^, 0.6 μm^2^–1.0 μm^2^ and >1.0 μm^2^.^26^ In support of the overall area and perimeter calculations, the analysis demonstrated that stress‐enhanced mitochondrial fission and reduced mitochondrial fusion with prolonged stress exposure, as evidenced by the obvious increase in the number of mitochondria with size smaller than <0.6 μm^2^ as well as a significant decrease those with size larger than >1.0 μm^2^ (Figure 2B,C). These changes include alterations in the expression of mRNAs and proteins involved in mitochondrial fission and fusion. anova revealed that the stress treatment had a significant effect on fission transcripts (Drp1: *F*_(4,16)_ = 38.89, *p* < 0.001; Fis1: *F*_(4,16)_ = 20.28, *p* < 0.001). Post hoc tests indicated that fission transcripts were significantly increased after 7 days (Drp1: *p* < 0.01; Fis1: *p* < 0.01) and 21 days (Drp1: *p* < 0.01; Fis1: *p* < 0.05) of stress exposure compared to the control group, although no difference was observed after 3 days (Drp1 and Fis1: *p* > 0.05) (Figure 2D,H). Changes in fission transcript expression were paralleled by similar effects at the protein level, as measured by western blotting (Figure 2E–G,I,J). anova also showed a significant effect of stress treatment on fission proteins (Drp1^ser616^: *F*_(4,16)_ = 22.04, *p* < 0.001; Fis1: *F*_(4,16)_ = 29.93, *p* < 0.001), although there were no significant changes in protein expression of Drp1 (*F*_(4,16)_ = 2.26, *p* > 0.05). Post hoc tests showed that fission proteins increased after 3 days (Drp1^ser616^: *p* < 0.05; Fis1: *p* < 0.05), 7 days (Drp1^ser616^: *p* < 0.01; Fis1: *p* < 0.01) and 21 days (Drp1^ser616^: *p* < 0.01; Fis1: *p* < 0.05) of stress exposure compared with the control group. In contrast to the increase observed in mitochondrial fission, anova showed that stress treatment had a significant inhibitory effect on fusion transcripts (Mfn1: *F*_(4,16)_ = 24.80, *p* < 0.001; Mfn2: *F*_(4,16)_ = 43.04, *p* < 0.001; Opa1: *F*_(4,16)_ = 13.32, *p* < 0.001) (Figure 2K,N,Q). Post hoc tests indicated that fusion transcripts were significantly decreased after 7 days (Mfn1: *p* < 0.01; Mfn2: *p* < 0.01; Opa1: *p* < 0.01) and 21 days (Mfn1: *p* < 0.05; Mfn2: *p* < 0.01; Opa1: *p* < 0.05) of stress exposure compared to the control group, although no difference was observed after 3 days (Mfn1, Mfn2, and Opa1: *p* > 0.05). In addition, a significant decrease in fusion proteins was found after stress treatment (Mfn1: *F*_(4,16)_ = 10.59, *p* = 0.001; Mfn2: *F*_(4,16)_ = 11.93, *p* < 0.001; Opa1: *F*_(4,16)_ = 17.70, *p* < 0.001). Post hoc tests showed that the fusion proteins were decreased after 3 days (Mfn1: *p* < 0.05; Mfn2: *p* < 0.05; Opa1: *p* < 0.05), 7 days (Mfn1: *p* < 0.01; Mfn2: *p* < 0.01; Opa1: *p* < 0.01) and 21 days (Mfn1: *p* < 0.01; Mfn2: *p* < 0.05; Opa1: *p* < 0.01) of stress exposure compared to the control group (Figure 2L,M,O,P,R,S). We also quantified Cyto‐c mRNA and protein levels in the mitochondria, which could reflect mitochondrial damage. As shown in Figure 2T, anova for cyto‐c mRNA showed significant effects of stress exposure (*F*_(4,16)_ = 24.24, *p* < 0.001). Post hoc tests showed that Cyto‐c mRNA significantly increased after 7 days (*p* < 0.01) and 21 days (*p* < 0.01) of stress exposure compared to the control group, although no difference was observed after 3 days (*p* > 0.05). anova also revealed a significant effect of stress on cytochrome c protein levels (F_(4,16)_ = 18.17, *p* < 0.001). Post hoc tests showed that Cyto‐c protein levels were significantly increased after 3 days (*p* < 0.05), 7 days (*p* < 0.01) and 21 days (*p* < 0.05) of stress exposure compared to the control group (Figure 2U,V). Mitochondrial damage disrupts ROS production and energy metabolism. Therefore, we evaluated ROS levels in cardiomyocytes. anova revealed significant changes in ROS levels after stress exposure (*F*_(3,12)_ = 96.31, *p* < 0.001) (Figure 3A,B). Post hoc tests indicated that intracellular ROS levels significantly increased after 3 days (*p* < 0.05), 7 days (*p* < 0.01) and 21 days (*p* < 0.01) of stress exposure compared to the control group.

### 3.3 Mitochondria dynamic disorder induced by stress‐activated endoplasmic reticulum stress JNK pathway

Owing to the close contact and information transmission between mitochondria and the endoplasmic reticulum, we further focused on the expression of mRNA and proteins related to endoplasmic reticulum stress. anova showed significant transcript changes of IRE1‐α (*F*_(4,16)_ = 24.17, *p* < 0.001), ASK1 (*F*_(4,16)_ = 18.65, *p* < 0.001) and JNK (*F*_(4,16)_ = 19.23, *p* < 0.001) (Figure 3C–E). Post hoc test indicated that transcripts of endoplasmic reticulum stress were significantly increased after 7 days (IRE1‐α: *p* < 0.01; ASK1: *p* < 0.01; JNK: *p* < 0.01) and 21 days (IRE1‐α: *p* < 0.01; ASK1: *p* < 0.01; JNK: *p* < 0.01) of stress exposure compared with the control group, whereas no difference was observed after 3 days (IRE1‐α, ASK1 and JNK: *p* > 0.05). We also identified a significant effect of stress on proteins changes of IRE1‐α (*F*_(4,16)_ = 15.78, *p* < 0.001), ASK1 (*F*_(4,16)_ = 28.59, *p* < 0.001), and JNK (*F*_(4,16)_ = 21.13, *p* < 0.001). As depicted Figure 3F–K, there was a significant increase in proteins of endoplasmic reticulum stress after 3 days (IRE1‐α: *p* < 0.01; ASK1: *p* < 0.01; JNK: *p* < 0.01), 7 days (IRE1‐α: *p* < 0.01; ASK1: *p* < 0.01; JNK: *p* < 0.01) and 21 days (IRE1‐α: *p* < 0.05; ASK1: *p* < 0.05; JNK: *p* < 0.05) of stress exposure compared to the control group.

### 3.4 Stabilisation of mitochondrial fusion and fission significantly improved mitochondrial morphology

**FIGURE 4:**
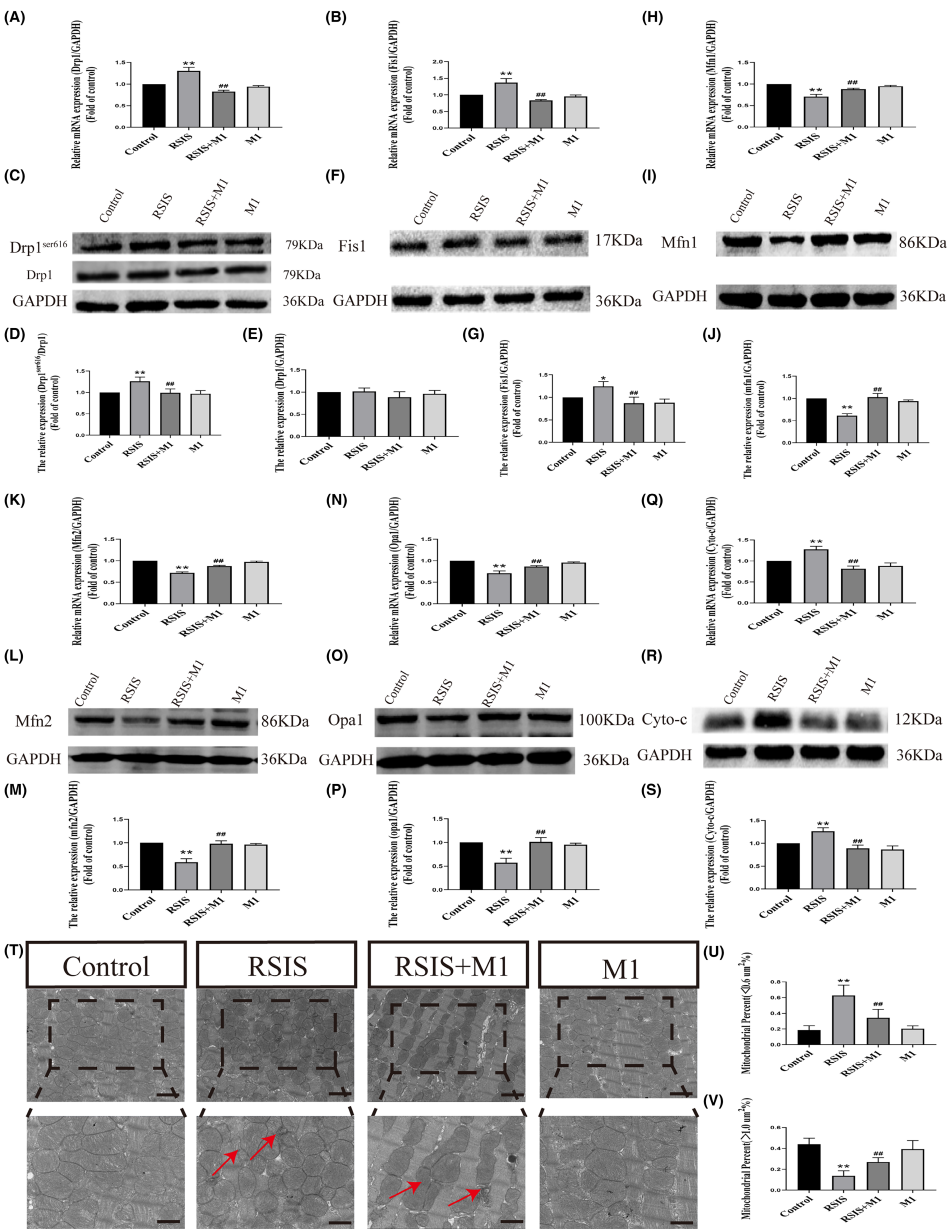
Stabilisation of mitochondrial fusion and fission (mitochondria fission, red arrows) significantly improved mitochondrial morphology. Scale bars = 2.0 μm or 1.0 μm. (A–S) Quantifying mRNAs and proteins of mitochondrial fission and fusion using Mdivi‐1 treatment. (T–V) Ultrastructural changes of mitochondria in cardiomyocytes. Mdivi‐1 significantly inhibited mitochondrial fission and increased mitochondrial fusion, and mitochondrial morphology was ameliorated. Data were presented as mean ± SD, *p < 0.05, **p < 0.01 compared with control, ^#^ *p* < 0.05, ^##^ *p* < 0.01 compared with RSIS, *n* = 4.

To investigate the effect of mitochondrial fusion and fission regulatory factors on mitochondrial morphology, we applied the Drp1 inhibitor Mdivi‐1 to stabilize mitochondrial fusion and fission. Two‐way anova of fission transcripts revealed significant effects on the interaction among stress combined with Mdivi‐1 treatment (Drp1: *F*_(1,16)_ = 83.30, *p* < 0.001; Fis1: *F*_(1,16)_ = 51.94, *p* < 0.001), stress treatment (Drp1: *F*_(1,16)_ = 17.43, *p* < 0.01; Fis1 *F*_(1,16)_ = 13.77, *p* < 0.01) and Mdivi‐1 treatment (Drp1: *F*_(1,16)_ = 137.56, *p* < 0.001; Fis1: *F*_(1,16)_ = 75.13, *p* < 0.001). After stress treatment, post hoc tests showed increased fission transcripts (Drp1 and Fis1: *p* < 0.01). In contrast, stress and Mdivi‐1 treatment significantly decreased the expression of fission transcripts (Drp1 and Fis1: *p* < 0.01) (Figure 4A,B). The changes in fission protein expression were consistent with the fission mRNA levels. Two‐way anova of fission proteins showed that the interaction of stress and Mdivi‐1 treatment, stress treatment alone had significant effects on the expression of Drp1^ser616^ (*F*_(1,16)_ = 9.63, *p* < 0.01; *F*_(1,16)_ = 13.66, *p* < 0.01) and Fis1 (*F*_(1,16)_ = 7.27, *p* < 0.05; *F*_(1,16)_ = 5.96, *p* < 0.05), while no significant effect on Drp1 (*F*_(1,16)_ = 1.23, *p >* 0.05; *F*_(1,16)_ = 0.50, *p >* 0.05). As shown in Figure 4C–G, the post hoc test showed that stress and Mdivi‐1 treatment resulted in decreased Drp1^ser616^ (*p* < 0.01) and Fis1 (*p* < 0.01) expression. We also investigated fusion mRNAs and protein expression of mitochondrial fusion proteins following Mdivi‐1 treatment and stress. As shown in Figure 4H,K,N, two‐way anova indicated significant effects of the interaction of stress and Mdivi‐1 treatment, stress treatment and Mdivi‐1 treatment alone on fusion mRNAs [(Mfn1: *F*_(1,16)_ = 58.91, *p* < 0.001; *F*_(1,16)_ = 150.57, *p* < 0.001; *F*_(1,16)_ = 17.81, *p* < 0.001); (Mfn2: *F*_(1,16)_ = 138.08, *p* < 0.001; *F*_(1,16)_ = 544.29, *p* < 0.001; *F*_(1,16)_ = 69.18, *p* < 0.001); (Opa1: *F*_(1,16)_ = 44.67, *p* < 0.001; *F*_(1,16)_ = 161.54, *p* < 0.001; *F*_(1,16)_ = 14.60, *p* < 0.001)]. After stress treatment, post hoc tests showed decreased fusion transcripts (Mfn1, Mfn2 and Opa1; *p* < 0.01). In contrast, Mdivi‐1 and stress treatments significantly increased the expression of the fusion mRNAs (Mfn1, Mfn2 and Opa1; *p* < 0.01). Similar effects were observed at the protein level. Two‐way anova showed that the interaction of stress and Mdivi‐1 treatment, stress treatment alone had significant effects on the protein expression of Mfn1 (*F*_(1,16)_ = 87.64, *p* < 0.001; *F*_(1,16)_ = 33.12, *p* < 0.001), Mfn2 (*F*_(1,16)_ = 72.79, *p* < 0.001; *F*_(1,16)_ = 61.28, *p* < 0.001) and Opa1 (*F*_(1,16)_ = 51.20, *p* < 0.001; *F*_(1,16)_ = 29.45, *p* < 0.001). As shown in Figure 4I,J,L,M,O,P, post hoc tests showed that stress and Mdivi‐1 treatment led to a significant increase in Mfn1 (*p* < 0.01), Mfn2 (*p* < 0.01) and Opa1 (*p* < 0.01) expression compared with stress treatment alone. We also investigated the cytochrome c mRNA and protein levels after Mdivi‐1 and stress treatments. Two‐way anova showed of Cyto‐c mRNA and protein revealed the main effects for stress treatment (mRNA: *F*_(1,16)_ = 10.76, *p* < 0.01; protein: *F*_(1,16)_ = 18.00, *p* < 0.01), Mdivi‐1 treatment (mRNA: *F*_(1,16)_ = 87.95, *p* < 0.001; protein: *F*_(1,16)_ = 56.11, *p* < 0.001), and a significant interaction of stress and Mdivi‐1 treatment (mRNA: *F*_(1,16)_ = 31.55, *p* < 0.001; protein: *F*_(1,16)_ = 12.09, *p* < 0.01) (Figure 4Q,R,S). Post hoc tests indicated that there was a significant difference in cyto‐c mRNA (*p* < 0.01) and protein (*p* < 0.01) levels between stress treatment alone and stress and Midiv‐1 treatment. These results indicated that Mdivi‐1 could significantly stabilize mitochondrial fusion and fission. We further observed the ultrastructural changes in the mitochondria of cardiomyocytes and calculated the area and perimeter of the mitochondria. As expected, mitochondrial fusion and fission stabilisation significantly improved mitochondrial morphology (Figure 4T–V).

### 3.5 Mitochondrial re‐homeostasis alleviated stressed myocardial injury by inhibition of endoplasmic reticulum stress JNK pathway

**FIGURE 5:**
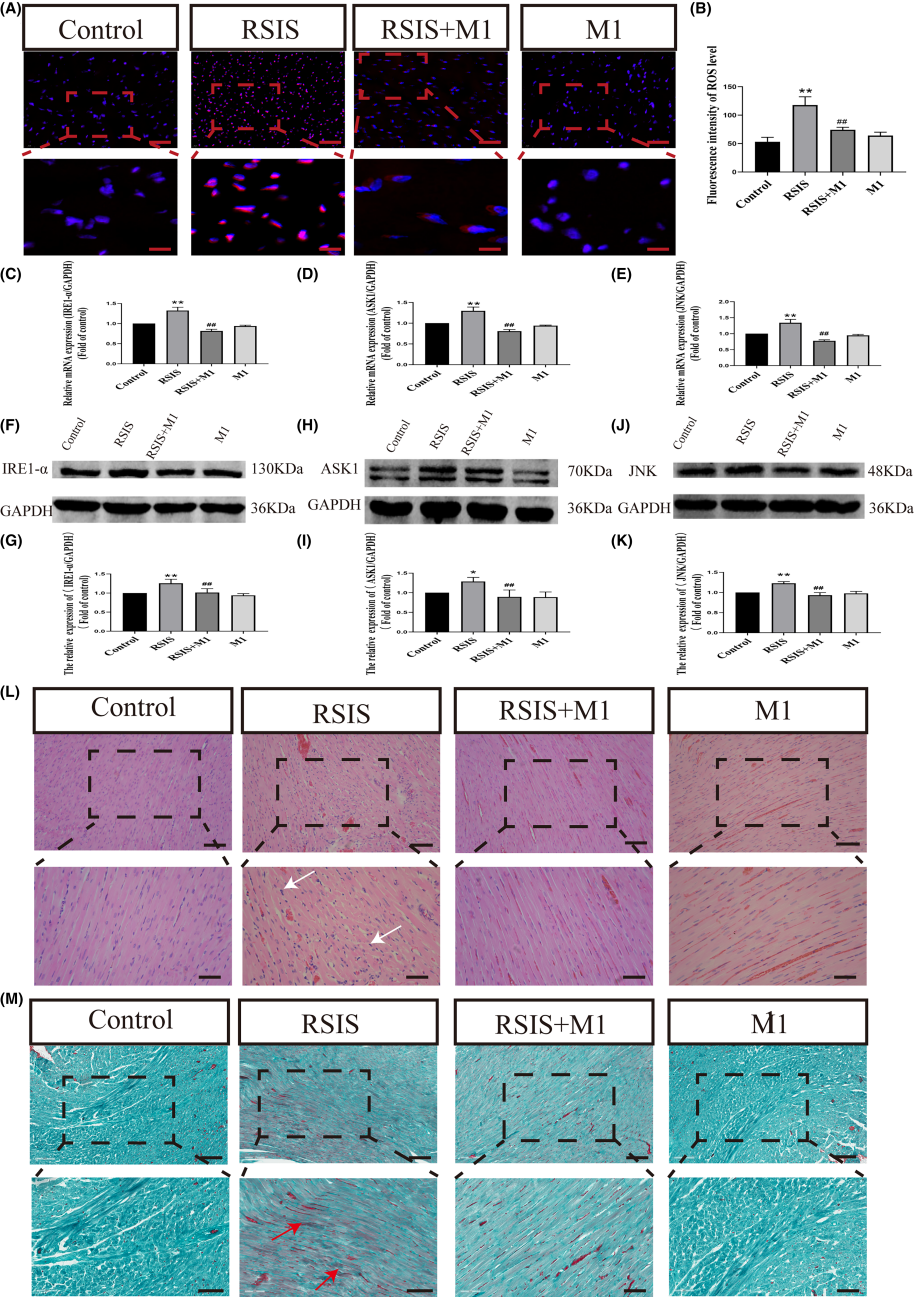
Mitochondrial re‐homeostasis alleviated stressed myocardial injury by inhibition of endoplasmic reticulum stress JNK pathway. (A, B) Quantification of intracellular ROS level after Mdivi‐1 treatment. Scale bars = 20 μm. (C–K) Quantification of mRNAs and proteins of endoplasmic reticulum stress JNK pathway after Mdivi‐1 treatment. Data were presented as mean ± SD, *p < 0.05, **p < 0.01 compared with control, ^#^ *p* < 0.05, ^##^ *p* < 0.01 compared with RSIS, *n* = 4. (L, M) Haematoxylin–eosin staining (inflammatory cells, white arrows) and Chromotrope‐2R brilliant green staining of heart after Mdivi‐1 treatment (ischemia and hypoxia, arrows). Scale bars = 100 μm or 50 μm.

Subsequently, we investigated the effects of mitochondrial rehomeostasis on ROS production. As shown in Figure 5A,B, Two‐way anova indicated obvious effects of the interaction between stress and Mdivi‐1 treatment and stress treatment alone on ROS levels (*F*_(1,12)_ = 26.69, *p* < 0.01; *F*_(1,12)_ = 50.56, *p* < 0.01). Post hoc tests indicated that stress and Mdivi‐1 treatment significantly decreased ROS levels (*p* < 0.01) compared to stress treatment alone. We further investigated the effect of the re‐balance of mitochondria on the endoplasmic reticulum stress JNK pathway. As shown in Figure 5C–E, two‐way anova indicated obvious effects of the interaction of stress and Mdivi‐1 treatment, stress treatment alone on mRNA expression of IRE1‐α (*F*_(1,16)_ = 85.71, *p* < 0.001; *F*_(1,16)_ = 17.59, *p* < 0.01), ASK1 (*F*_(1,16)_ = 75.34, *p* < 0.001; *F*_(1,16)_ = 10.52, *p* < 0.01) and JNK (*F*_(1,16)_ = 79.61, *p* < 0.001; *F*_(1,16)_ = 8.69, *p* < 0.05). Post hoc test indicated that stress and Mdivi‐1 treatment led to a significant decrease in IRE1‐α (*p* < 0.01), ASK1 (*p* < 0.01) and JNK (*p* < 0.01) mRNA expression compared to stress treatment alone. In addition, two‐way anova of endoplasmic reticulum stress proteins also showed that the interaction of stress and Mdivi‐1 treatment, stress treatment alone had significant effects on the expression of IRE1‐α (*F*_(1,16)_ = 5.47, *p* < 0.05; *F*_(1,16)_ = 17.77, *p* < 0.01), ASK1 (*F*_(1,16)_ = 5.24, *p* < 0.05; *F*_(1,16)_ = 5.86, *p* < 0.05) and JNK (*F*_(1,16)_ = 35.99, *p* < 0.001; *F*_(1,16)_ = 15.79, *p* < 0.01) (Figure 5F–K). Post hoc test indicated that stress and Mdivi‐1 treatment resulted in a significant decrease in IRE1‐α (*p* < 0.01), ASK1 (*p* < 0.01) and JNK (*p* < 0.01) expression compared with those in stress treatment. Re‐balance of mitochondria effectively alleviates endoplasmic reticulum stress. We further observed morphological changes in cardiomyocytes using HE and Chromotrope‐2R brilliant green staining. These results indicated that mitochondrial re‐homeostasis improved stress myocardial injury (Figure 5L,M).

## 4 DISCUSSION

Stress refers to a series of neuroendocrine, physiological and pathological reactions that occur when the body is stimulated by various stressors (e.g., somatic and psychological). Stress promotes the release of stress hormones by activating the HPA and locus coeruleus–sympathetic–adrenomedullary axes, which regulate metabolism, immunity, digestion and other processes to maintain homeostasis. However, intense and (or) prolonged stress can lead to excessive release of catecholamines and glucocorticoids, which cause damage to the heart. Previous studies have indicated that excess norepinephrine can activate norepinephrine receptors rich on the myocardial surface, leading to inadequate myocardial blood supply, calcium overload and oxidative stress, which are involved in myocardial injury.^2^, ^26^, ^27^ However, the exact molecular mechanisms underlying this injury remain unclear. To further investigate the role of stress in cardiomyocyte injury, we used a classical rat model characterized by somatic and psychological stress to simulate the effects of prolonged stress on the human heart. Consistent with previous studies, we found that cardiomyocytes were injured by prolonged stress using biochemical index assays and observation of cardiac morphology changes.

A critical aspect of cardiac function maintenance is requiring much energy from mitochondria. Mitochondria are highly dynamic organelles responsible for synthesising the energy‐carrying molecule adenosine triphosphate (ATP) and regulating power‐related cellular processes in the heart.^28^, ^29^ Previous studies have shown that more than 40% of the cytoplasmic space of adult cardiomyocytes is occupied by a large number of mitochondria,^31^ which are mainly located between the sarcomere, around the nucleus and under the plasma membrane of cardiomyocytes,^31^ and continuously supply energy for myocardial contraction. Through electron microscopic observation and analysis, we found that stress reduced the area and circumference of mitochondria in cardiomyocytes, suggesting that mitochondrial fragmentation occurred and mitochondria were damaged.

Mitochondria are stress‐sensitive organelles, and their morphology and function are closely related. Mitochondria regulate their morphology under continuous fusion and fission to meet cell energy and metabolic requirements.^11^, ^31^ Mitochondrial fusion is regulated by three GTPase‐dependent proteins, namely Mfn1 and Mfn2, which regulate outer membrane fusion, and OPA1, which regulates inner membrane fusion. Accumulating evidence indicates that deletion of Mfn1 and Mfn2 causes abnormal mitochondrial structure,^32^, ^33^ whereas Opa1 deletion mainly affects endosomal cristae formation, leading to impaired mitochondrial energy production.^34^, ^35^ Mitochondrial fission is mediated by Drp1, which oligomerically contracts and cleaves the mitochondrial membrane by binding to the ligand Fis1 of the outer mitochondrial membrane after phosphorylation at serine 616.^27^ To verify the relationship between mitochondrial fragmentation, mitochondrial fusion and fission, we investigated the key markers of mitochondrial fusion and fission at the transcriptional and protein levels. These results showed that stress significantly inhibited mitochondrial fusion and increased mitochondrial fission, indicating that mitochondrial fragmentation in cardiomyocytes is caused by stress‐induced turbulence of mitochondrial fusion and fission. Aberrant expression of these key proteins can induce activation of the mitochondrial permeability transition pore (MPTP)^16^, ^36^ and increase permeabilisation of the outer membrane (MOMP),^38^ leading to the release of Cyto‐c. Therefore, with prolonged stress exposure, the expression of Cyto‐c significantly increased, which reflected mitochondrial dysfunction.

As broadly documented in the literature, there are structural and functional contacts between mitochondria and other organelles, including mitochondrial endoplasmic reticulum contacts, namely, mitochondria‐associated endoplasmic reticulum membranes.^38^, ^39^ Under various physiological and pathological conditions, mitochondria transmit ion, lipid or protein signals to the endoplasmic reticulum through this membrane.^40^, ^41^ Some studies have suggested that cellular stress disturbs information transmission between the mitochondria and endoplasmic mainly by activating the endoplasmic reticulum stress response.^45^ Stress‐induced turbulence in mitochondrial fusion and fission in cardiomyocytes can trigger the production of reactive oxygen species, energy metabolism dysfunction and calcium overload. After stress exposure, we evaluated ROS production in cardiomyocytes and found that mitochondrial dysfunction increased ROS levels. Enhanced ROS production is a critical trigger of endoplasmic reticulum stress. Therefore, we evaluated the proteins related to endoplasmic reticulum stress and demonstrated that increased ROS production affected signal transmission between the mitochondria and endoplasmic and activated the endoplasmic reticulum stress. During endoplasmic reticulum stress, IRE1α has a trans‐autophosphorylated and RNase domain. IRE1‐α's RNase activated the excised intron of the encoded transcription factor XBP1 to form active XBP1s. IRE1α‐XBP1 regulated genes enhance protein folding and translocation and promote protein degradation to relieve protein load in the endoplasmic reticulum. In addition, IRE1α activation also mediates mRNA attenuation to reduce the misfolded protein load on the endoplasmic reticulum, called IRE1‐dependent regulatory attenuation (RIDD). Ultimately, IRE1‐α continuously recruits TNF receptor‐associated factor 2 (TRAF2), apoptosis signal‐regulated kinase 1 (ASK1) and its downstream target JNK, and JNK overexpression promote cell death.^43^, ^44^ Therefore, we hypothesized that the endoplasmic reticulum stress in the JNK signalling pathway, triggered by disrupted mitochondrial fusion and fission, is an important cause of stress‐induced cardiomyocyte injury. To further verify this hypothesis, we used the mitochondrial fission inhibitor, Mdivi‐1, to enhance mitochondrial stability. As expected, Mdivi‐1 significantly inhibited mitochondrial fission, increased mitochondrial fusion, ameliorated mitochondrial morphology and decreased ROS production. Previous studies have shown that Mdivi‐1 attenuates the turbulence of mitochondrial fusion, fission and endoplasmic reticulum stress, improving pulmonary artery smooth muscle cell (PASMC) function in vitro and in vivo.^45^, ^46^ In our experiments, the data also indicated that using Mdivi‐1 effectively alleviated endoplasmic reticulum stress and stress‐induced cardiac injury pathomorphological changes, such as reduction of inflammatory cell infiltration and the area of ischemic and hypoxic damage. Although the effect of Mdivi‐1 is directly related to the endoplasmic reticulum, which is not yet clear, it has been shown that Mdivi‐1, which stabilizes mitochondrial fusion and fission, plays a protective role against stress‐induced cardiac injury. Therefore, our results confirmed that endoplasmic reticulum stress induced by the turbulence of mitochondrial fusion and fission is involved in stressed cardiomyocyte injury. Many people have been suffering from somatic and(or) psychological stress, and stress‐induced heart health issues remain a significant challenge. Our study suggests that stress‐induced cardiomyocyte injury by impairing mitochondrial fusion/fission homeostasis and activating reticulum IRE1/ASK1/JNK signalling may be a potential therapeutic target for preventing stress‐related myocardial injury; however, the exact molecular mechanisms need to be further investigated.

## AUTHOR CONTRIBUTIONS

**Shengnan Zhang:** Methodology (equal); supervision (equal). **Yingmin Li:** Methodology (equal); supervision (equal). **Weihao Zhu:** Data curation (equal). **Lihua Zhang:** Software (equal). **Lei Lei:** Software (equal). **Xiaofei Tian:** Software (equal). **Ke Chen:** Software (equal). **Weibo Shi:** Validation (equal). **Bin Cong:** Writing – review and editing (equal).

## FUNDING INFORMATION

This work was supported by the Key Projects of the National Natural Science Foundation of China (82130055), National Natural Science Foundation of China (82072109 and 81971787) and the Key Research and Development Projects in Hebei Province (20377722D).

## CONFLICT OF INTEREST STATEMENT

The authors declare no conflicts of interest.

## Supporting information


Data S1:

## Data Availability

The data that support the findings of this study are available from the corresponding author upon reasonable request.
