# Supplementary material for: Endoplasmic reticulum stress induced by turbulence of mitochondrial fusion and fission was involved in stressed cardiomyocyte injury
Source: J Cell Mol Med. 2023 Aug 18;27(21):3313–25. doi: 10.1111/jcmm.17901 (PMC10623534; doi:10.1111/jcmm.17901)
Supplement: Supplementary file 1 — Data S1: [file JCMM-27-3313-s001.docx]

**SUPPLEMENTARY MATERIALS AND METHODS**

**Serum** **norepinephrine test**

Additionally, the rats were anesthetized with 10% chloral hydrate intraperitoneally, and blood was collected by cardiac puncture. The samples were centrifuged at 4℃, 1000 ×g for 15 min, and the supernatant was collected and stored at -80℃. ELISA kits were used to measure the content of serum norepinephrine (NE) (Hcusabio, CSB-E07022r, Wuhan, China) in different groups. According to the manufacturer's instructions, and norepinephrine levels were determined using a rabbit-derived NE ELISA kit. Briefly, setting up standard wells and sample wells, 50μL of standards of different concentrations are to be added to each of the standard wells, and 50μL of the sample is to be added to the sample wells; blank wells are not added, except for the blank wells, 100μL of HRP-labeled detection antigen to be added each of the standard and sample wells, and incubated the wells in a 37℃ thermostat for one hour. They were then washed five times with washing buffer. Subsequently, each well-added 50μl of substrate A and B was incubated for 15 min at 37°C, avoiding light. Add 50μL The termination solution to each well, and the OD value of each well was measured at 450 nm within 15 min.

**Elevated plus-maze (EPM)**

The EPM consisted of two open arms (50 × Χ10 cm), two closed arms (50 × Χ10 × Χ40 cm), and a central area (10 × Χ10 cm). The Plexiglass arms were elevated 50 cm above the ground. Behavioral testing was performed under dim red light (28 lx). Each rat was recorded using an overhead camera, and its behavior was scored for 5 min using an automated video tracking system (ANY-maze v.4.6, Stoelting, Wood Dale, IL, USA). The percentage of time spent in the open arms {[time in open arms/ (time in open arms + time in closed arms)]Χ100} and the percentage of open arm entries{[open arm entries/(open arm entries +closed arm entries)]Χ100} were used as measures of anxiety-like behavior, and the number of closed arm entries was used as a measure of general locomotor activity.

**SUPPLEMENT RESULTS**

**Stress caused the changes in rat body weight, NE content, rat behaviors, and stool count**

These changes are physiological indicators of stress. As shown in supplement figure 1A, the weight of rats in the control group showed a significant increase after 3d (228.42±9.25), 7d (252.33±6.92), and 21d (275.42±15.58). In comparison, the weight of stress rats slightly decreased after 3d (199.25±5.89) and slowly increased after 7d (202.75±7.23) and 21d (240.25±9.93) stress exposure. ANOVA of the NE content showed significant changes after stress exposure (*F_(4, 16)_*=10.14, *P* < 0.001). *Post hoc* comparisons showed that NE levels significantly increased after 3d (*P* < 0.05), 7d (*P* < 0.01), and 21d (*P* < 0.05) of stress exposure (Figure 1B). For the EPM, the ANOVA revealed that stress treatment led to significant differences in the percentage of time spent in the open arms ( *F_(6, 24)_*=17.83, *P* < 0.001) and the percentage of open arm entries ( *F_(6, 24)_*= 48.67, *P* < 0.001). *Post hoc* tests indicated that the percentage of time spent in the open arms and the percentage of open-arm entries were markedly lower in the stress groups (*P* < 0.01, *P* < 0.01) (Figure 1C, D). ANOVA of the stool count results showed significant differences after stress exposure (*F_(6, 24)_*=10.53, *P* < 0.001). Compared with the control group, the stool count was markedly increased in the stress group at 3 (*P <*0.05), 7 (*P <*0.01), and 21d (*P <*0.01) (Figure 1E).


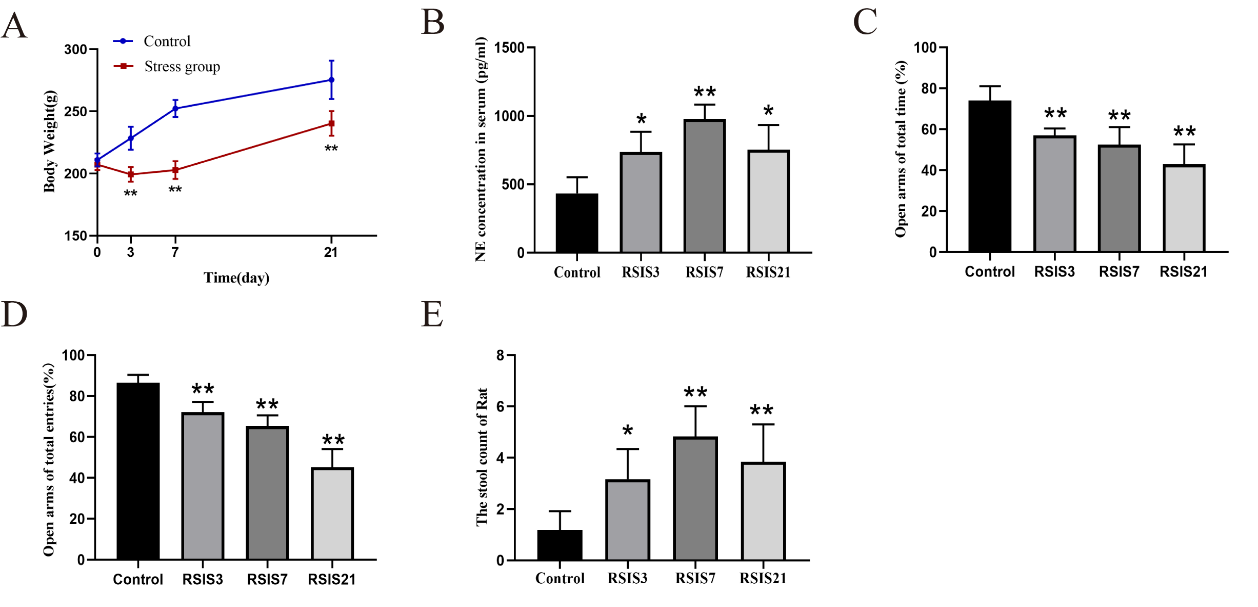


Supplementary Fig 1: Stress-induced changes of body weight and anxiety-behavior of rats. (A) Body weight changes after stress exposure. (B) Serum NE changes after stress exposure. (C) EPM ratio for Open arms time / Total time among different groups. (D) EPM ratio for Open arms entries / Total entries among different groups. (E) The number of stool counts of rats. Data are presented as mean ± SD, n=12 for body weight, and n=6 for EPM analysis. ^*^*P*＜0.05，^**^*P*＜0.01 compared with control. There was an insignificant difference between the control group with time (not shown).
